# Skeletal muscle volume following dehydration induced by exercise in heat

**DOI:** 10.1186/2046-7648-1-3

**Published:** 2012-09-04

**Authors:** Kyle J Hackney, Summer B Cook, Timothy J Fairchild, Lori L Ploutz-Snyder

**Affiliations:** 1 Department of Exercise Science, Syracuse University, 820 Comstock Ave, Room 201Women’s Building, Syracuse, NY, 13244, USA; 2 Present address: Wyle Integrated Science and Engineering, Exercise Physiology and Countermeasures Project, Houston, TX, 77058, USA; 3 Department of Kinesiology, University of New Hampshire, 124 Main Street, Durham, NH, 03824, USA; 4 School of Chiropractic and Sports Science, Murdoch University, 90 South Street, Murdoch, WA, 6150, Australia; 5 Universities Space and Research Association, Exercise Physiology and Countermeasures Project, NASA Lyndon B Johnson Space Center, 2101 NASA Parkway, SK/261, Houston, TX, 77058, USA

**Keywords:** Dehydration, Skeletal muscle, MRI, Cycling, Total body water, Fluid shift

## Abstract

**Background:**

Intracellular skeletal muscle water is redistributed into the extracellular compartment during periods of dehydration, suggesting an associated decline in muscle volume. The purpose of this study was to evaluate skeletal muscle volume in active (knee extensors (KE)) and less active (biceps/triceps brachii, deltoid) musculature following dehydration induced by exercise in heat.

**Methods:**

Twelve participants (seven men, five women) cycled in the heat under two conditions: (1) dehydration (DHYD) resulting in 3% and 5% losses of estimated total body water (_E_TBW), which was assessed by changes in body mass, and (2) fluid replacement (FR) where 3% and 5% losses of _E_TBW were counteracted by intermittent (20 to 30 min) fluid ingestion via a carbohydrate-electrolyte beverage. During both conditions, serum osmolality and skeletal muscle volume (assessed by magnetic resonance imaging) were measured at baseline and at the 3% and 5% _E_TBW loss measurement points.

**Results:**

In DHYD, serum osmolality increased at 3% (*p* = 0.005) and 5% (*p* < 0.001) _E_TBW losses, while FR decreased serum osmolality at the 5% loss of _E_TBW time point (*p* = 0.009). In DHYD, KE muscle volume declined from 1,464 ± 446 ml to 1,406 ± 425 ml (3.9%, *p* < 0.001) at 3% _E_TBW loss and to 1,378 ± 421 ml (5.9%, *p* < 0.001) at 5% _E_TBW loss. The largest decline in KE volume in DYHD occurred in the mid-belly (31 ml, p = 0.001) and proximal (24 ml, *p* = 0.001) regions of the grouped vasti muscles. There were no changes in volume for the biceps/triceps (*p* = 0.35) or deltoid (*p* = 0.92) during DHYD. FR prevented the loss of KE muscle volume at 3% (1,430 ± 435 ml, *p* = 0.074) and 5% (1,431 ± 439 ml, *p* = 0.156) _E_TBW loss time points compared to baseline (1,445 ± 436 ml).

**Conclusions:**

Following exercise in the heat, the actively contracting muscles lost volume, while replacing lost fluids intermittently during exercise in heat prevented this decline. These results support the use of muscle volume as a marker of water loss.

## Background

Water accounts for 50% to 60% of the total body mass [1] and approximately 75% of the muscle mass [2]. A loss of total body water (TBW) equivalent or greater than 2% of body mass can significantly reduce performance on prolonged submaximal tasks [2,3] and impaired muscular strength and power [4]. Interestingly, the extent of water loss from specific tissue compartments including the skeletal muscle is not well understood and may depend on the means through which dehydration is induced. In thermally dehydrated (approximately 10% body weight) rats, approximately 40% of the decline in TBW was attributed to intracellular stores within the skeletal muscle tissue [5]. This redistribution of water across the muscle cell membrane during dehydration is primarily dependent on the osmotic gradient [2] and the activity of ionic pumps [6]. However, when exercise is combined with heat to induce dehydration, the physiological and metabolic changes are likely to affect the mobilization of water from the skeletal muscle tissue. For example, the redistribution of blood flow to the active muscle during exercise [7] may account for up to 50% of the change in muscle size [8]. The accumulation of metabolites associated with energy-yielding pathways is known to alter the osmotic gradient [9] and will therefore result in water movement across tissue compartments, including both active and inactive muscle tissue compartments. Additionally, during prolonged exercise in the heat, there is an increase in plasma and serum osmolality [10,11], which resulted from a greater net loss of water as compared with sodium (Na+). This elevation in osmolality is the primary hypothesized mechanism for the mobilization of water across the muscle cell membrane [12]. Consumption of water or a carbohydrate-electrolyte beverage during prolonged exercise counteracts this increase in serum osmolality [10,11], thus negating one of the primary mechanisms whereby water is sequestered across the muscle cell membrane. The implications of fluid replacement via water or carbohydrate-electrolyte beverages on muscle water during exercise in heat are currently not known.

Storage of glycogen is also associated with water storage, and therefore, the concentration of glycogen will directly influence total muscle water [13]. Approximately 2.7 g of water are bound per gram of glycogen [14], and as glycogen concentration decreases, the water bound to glycogen is released [15]. Indeed, Costill et al. [16] demonstrated that both glycogen and water content in muscle fibers from the vastus lateralis were significantly reduced approximately 30 min after exhaustive cycling in heat. This corresponded to a rate of active skeletal muscle water decline of 1.2% for each 1% decrease in body weight. While no comparisons with less active muscles (e.g., upper body) were available in the study of Costill et al. [16], findings from others suggest that there is some glycogen breakdown occurring in less active tissues during exhaustive bouts of exercise [17].

Magnetic resonance imaging (MRI) offers outstanding spatial resolution of muscle anatomy including the ability to noninvasively evaluate the volume of individual muscles and/or muscle groups [18,19]. The application of MRI to assess changes in hydration status within the skeletal muscle tissue has not previously been reported in the exercise literature. Therefore, the purpose of this study was to evaluate the skeletal muscle volume in active and less active musculature during dehydration induced by exercise in heat. We hypothesized that (1) skeletal muscle volume would decline in both active and less active muscles during dehydration and (2) fluid replacement during exercise in heat would prevent a loss of skeletal muscle volume.

## Methods

### Participants

Twelve recreationally active men (*n* = 7) and women (*n* = 5) were recruited for the investigation (mean ± standard deviation (SD): body mass = 69.8 ± 16.4 kg, body fat = 17.2 ± 7.9%, estimated TBW (_E_TBW) = 40.0 ± 10.1 kg). Prior to participation, all subjects were screened for sickle cell trait (AccuBase A1c^TM^, Diabetes Technologies, Inc, Thomasville, GA, USA) due to the risk of microvascular occlusion during physical exertion [20]. Pregnant females were also excluded from participation due to possible risk of severe dehydration, and for this reason, a urine pregnancy test was adminstered to all female participants on the day of testing. Study participation was supervised and approved by a physician. The project was approved by the Institutional Review Boards from Syracuse University, SUNY Upstate Medical University, and the US Army Surgeon General. All participants provided their written informed consent prior to participating in the study.

### Preliminary testing

Prior to experimental testing, an estimate of stable baseline euhydrated body mass (nearest hundredth of 1 kg) was determined for each subject from mean values obtained over consecutive days (9.3 ± 0.8 days). Each measurements were obtained semi-nude using a calibrated digital scale (WeighSouth WSI-600, Mettler-Toledo, Inc, Worthington, OH, USA). These measurements were conducted at the same time of day in the laboratory after voiding and prior to eating. Fat and fat-free body mass were also determined via Dual Energy X-ray Absorptiometry (DEXA) (Lunar DPX; GE Medical Systems, Waukesha, WI, USA). Using the fat and fat-free mass from DEXA, _E_TBW was calculated using the following equation: _E_TBW = 0.10 kg (DEXA fat mass) + 0.73 kg (DEXA fat − free mass) [13,21]. During this time period, subjects also completed three to six acute, submaximal heat familiarization sessions (20 to 30 min) on a cycle ergometer in the climate chamber. Subjects performed familiarization sessions until a stable steady state heart rate was observed during submaximal exercise in the heat (data not shown) and until they felt they were comfortable with the testing protocol. Following stable body weight assessment and familiarization, subjects completed an experimental exercise in heat testing sessions under two conditions: (1) dehydration (DHYD) and (2) fluid replacement (FR).

### Baseline testing

Both DHYD and FR testing sessions were counterbalanced and began the morning (0700 hours) following an overnight fast (>8 h). On arrival to the laboratory, subjects voided their bladders for assessment urine specific gravity via a refractometer (PAL-10S, Atago, Belevue, WA, USA). Urine specific gravity was only evaluated at baseline to document that subjects were not dehydrated prior to baseline data collection. Body mass was measured using the digital scale previously described (0715 hours), and participants rested in the supine position of 30 min. Blood samples (approximately 5 ml) were obtained via arm venipuncture using Vacutainer® (BD, Franklin Lakes, NJ, USA) containing a clot activator and gel for serum separation (0745 hours). All blood samples were immediately transported to SUNY Medical University Hospital for clinical analysis of serum osmolality (Advanced® Model 330 Micro-Osmometer, Advanced Instruments, Inc., Norwood, MA, USA). Once blood samples were obtained, subjects were transported to the MRI scanner in the supine position using a gurney to avoid fluid shifts [22]. Once correctly positioned in the MRI scanner (0800 hours), 5-mm-thick transaxial images (2,122-ms repetition time, 0.5-mm slice-to-slice interval) were acquired using a 1.5-T Phillips Intera whole body scanner with the software Release 11 (Phillips Medical Systems, Bothell, WA, USA). MRI scans were obtained along the length of the right upper leg from the head of the femur to the knee and along the right upper arm between the head of the humerus to the elbow. Once baseline data collection was completed (0900 hours), participants walked to the climate chamber to complete one of the counterbalanced exercise and heat testing sessions.

### DHYD protocol

During the DHYD testing condition, participants cycled at 60 rpm on a Monark cycle ergometer in a climate chamber at 38°C, 32% humidity, and 13 km/h wind. Participants began pedaling at 1 kp but were allowed to change the cycle load if needed to achieve the desired _E_TBW loss. Rectal temperature (YSI 402AC, YSI Inc., Dayton, OH, USA) and heart rate (Polar A5, Polar Electro Inc., Lake Success, NY, USA) were monitored throughout the exercise sessions. During the DHYD exercise in heat session, no fluids were provided. Participants exercised in heat and were weighed every 20 to 30 min until _E_TBW loss reached 3% as assessed by body mass. At this time, subjects were allowed to void their bladders and walk to the resting area where a final weight was recorded. Subjects rested in the supine position until a blood sample and MRI scan was obtained 45 min and 60 min, respectively, after exercise. After dependent measures were obtained, the participants went back to the climate chamber for additional exercise in heat. No fluids were provided during acquisition of dependent measures; therefore, participants cycled in heat and were weighed until an additional 2% _E_TBW was lost (which equaled 5% _E_TBW loss total for the condition). Once 5% _E_TBW loss was achieved, the dependent measures (body mass, blood sample, and MRI) were obtained as described previously.

### FR protocol

The FR condition was identical to the DHYD condition except that all fluids lost during exercise and heat were intermittently (20 to 30 min) replaced at 1.5 times the rate they were lost using a carbohydrate-electrolyte beverage (14 g carbohydrate, 200 mg sodium, 90 mg potassium/8 oz; Gatorade® Endurance, Chicago, IL,USA) [23]. Replacement of 1.5 times the rate of fluid loss was selected in order to account for continuous sweat and urine production while dependent measures were assessed. Participants began by cycling in heat under the same intensity and thermal conditions previously described. Following 20 to 30 min of exercise in heat, the participants were removed from the thermal chamber and weighed to determine _E_TBW. This amount was recorded, and fluids were provided. Participants were then re-weighed with the consumed fluid and returned to the thermal chamber for an additional 20 to 30 min of exercise in heat. This process was repeated until the total amount of fluid lost after being removed from the thermal chamber totaled 3% _E_TBW loss. At this point, our goal was to have adequately replaced the 3%_E_TBW fluid, so there was no change in body mass. Participants were allowed to void their bladders and walk to the resting area where a final weight was recorded and dependent measures were assessed. Participants then returned to the heat chamber and exercised, and every 20 to 30 min, they were then weighed and given the appropriate amount of fluids. This process continued until an additional 2% _E_TBW was lost (then replaced); hence, the total amount of fluid lost then replaced was equivalent to 5% _E_TBW. At this point, the goal was to have no change in body mass from the baseline despite sweating out 5% _E_TBW during the exercise and heat session. After the 5% _E_TBW loss, time point was reached in FR, a final body mass was recorded, and dependent measures were reassessed.

### MRI analysis

Following both DHYD and FR conditions, MRI images were transferred to a computer for tracing, and skeletal muscle volume was calculated using the NIH ImageJ analysis software (NIH, Bethesda, MD, USA) [24]. Knee extensor muscle volume was determined by adding the volumes from the rectus femoris and vasti muscles, which were analyzed between the appearances of the distal portion of the rectus femoris and the femoral neck. The number of slices analyzed across the distance of the knee extensors was subsequently divided into three sections to determine regional changes in volume. The distal region was characterized by the first third of slices (toward the knee), the mid-belly represented the middle third of slices (mid-thigh), and the proximal region comprised the upper third of slices (toward the hip). Shoulder muscle volume was determined from the sum of five to six axial slices superior to the appearance of the anterior deltoid. Elbow flexor and extensor muscles were evaluated from the combined area of the biceps brachii and triceps brachii using the sum of five to six axial slices distal to the appearance of the anterior deltoid. The same number of slices was measured for each subject at each of the testing time points, and great care was taken to ensure within-subject measurement replication. Three investigators were responsible for MRI analysis (one for each muscle/group: knee extensors, biceps/triceps, deltoid). All MRI analyzers had previously demonstrated test-retest reliability of <1% in the laboratory and were blinded to condition and time point of the images.

### Statistical analysis

Urine specific gravity, chamber temperature, relative humidity, duration of chamber exposure, exercise workload, exercise heart rate, and core body temperature were compared between DHYD and FR conditions using paired *t* tests. Dependent measures of body mass, serum osmolality, and skeletal muscle volume were analyzed using within-subject analysis of variance (ANOVAs) with repeated measures on condition (DHYD, FR) and time (baseline, 3% _E_TBW loss, 5% _E_TBW loss). When significant condition × time interactions were determined, differences within and between conditions were evaluated using Bonferroni corrected Student's *t* tests. Regional (distal, mid-belly, proximal) changes in muscle volume were also evaluated *post hoc* using ANOVA with repeated measures on the muscle (grouped vasti muscles, rectus femoris) and time (baseline, 5% _E_TBW loss) with follow-up Bonferroni corrected Student's *t* tests. Associations among dependent variables were further explored using Pearson's correlations. MRI image quality (motion artifact) at one time point prevented the analysis of muscle volume on one male subject for the knee extensors and one female subject for the arm muscles. Therefore, both leg and arm MRI statistical analyses were performed using *n* = 11. Significance was determined at *p* < 0.05. All values are mean ± SD.

## Results

Baseline urine specific gravity did not differ statistically (*p* = 0.42) and were 1.016 ± 0.01 and 1.018 ± 0.01 for DHYD and FR conditions, respectively. Chamber temperature, humidity, exercise workload, and core temperature were closely matched between conditions (Table 1). In DHYD, the total climate chamber exposure time was significantly less, and heart rate was elevated compared with that in FR (Table 1).

**Table 1:** Environmental conditions and physiological responses

**Variable**	**DHYD**	**FR**	***p*value**
Chamber temperature (°C)	36.9 ± 0.6	37.3 ± 1.0	0.20
Chamber relative humidity (%)	32.4 ± 5.9	30.5 ± 5.9	0.22
Chamber exposure (min)	134 ± 31^†^	154 ± 29	0.02
Exercise workload (W)	96 ± 39	98 ± 40	0.67
Heart rate (beats min^−1^)	147 ± 11^†^	137 ± 7	0.01
Core body temperature (°C)	38.1 ± 0.6	38.2 ± 0.4	0.14

^†^Significantly different from FR, *p* < 0.05. *n* = 12; mean ± SD.

There was a significant condition × time interaction for body mass (*p* < 0.001, Table 2). At baseline, there were no differences between conditions (*p* = 0.16). Body mass was significantly lower in the DHYD compared with that in the FR at 3% (*p* = 0.007) and 5% (*p* < 0.001) _E_TBW loss measurement time points. Overall, _E_TBW loss at 3% and 5% measurement points were 3.45 ± 0.73% (*p* < 0.001) and 5.75 ± 0.86% (*p* < 0.001) in DHYD and 0.78 ± 0.81% (*p* < 0.05) and 0.64 ± 0.79% (*p* < 0.05) in FR, respectively.

**Table 2:** Body mass (kilograms) and serum osmolality (milliosmolar per kilogram)

**Variable**	**Condition**	**Baseline**	**↓3%**_**E**_**TBW**	**↓5%**_**E**_**TBW**
Body mass	DHYD	69.4 ± 16.2	67.9 ± 15.8*^†^	67.1 ± 15.6*^†^
	FR	69.1 ± 16.1	68.7 ±15.8*	68.8 ± 15.9*
Serum osmolality	DHYD	291 ± 7	304 ± 6*^†^	301 ± 4*^†^
	FR	296 ± 7	292 ± 4	290 ± 5*

*Significantly different within condition vs baseline, *p* < 0.05; ^†^significantly different from FR, *p* < 0.01. *n* = 12; mean ± SD.

There was a significant condition × time interaction for serum osmolality (*p* < 0.001, Table 2). At baseline, serum osmolality was similar between conditions (*p* = 0.76). In DHYD, osmolality increased at the 3% (*p* = 0.005) and 5% (*p* < 0.001) _E_TBW loss. In FR, serum osmolality decreased only at the 5% (*p* = 0.009) _E_TBW loss measurement time point. Serum osmolality was significantly greater in the DHYD condition compared with that in FR at both the 3% (*p* < 0.001) and 5% (*p* < 0.001) _E_TBW losses.

**Figure 1:**
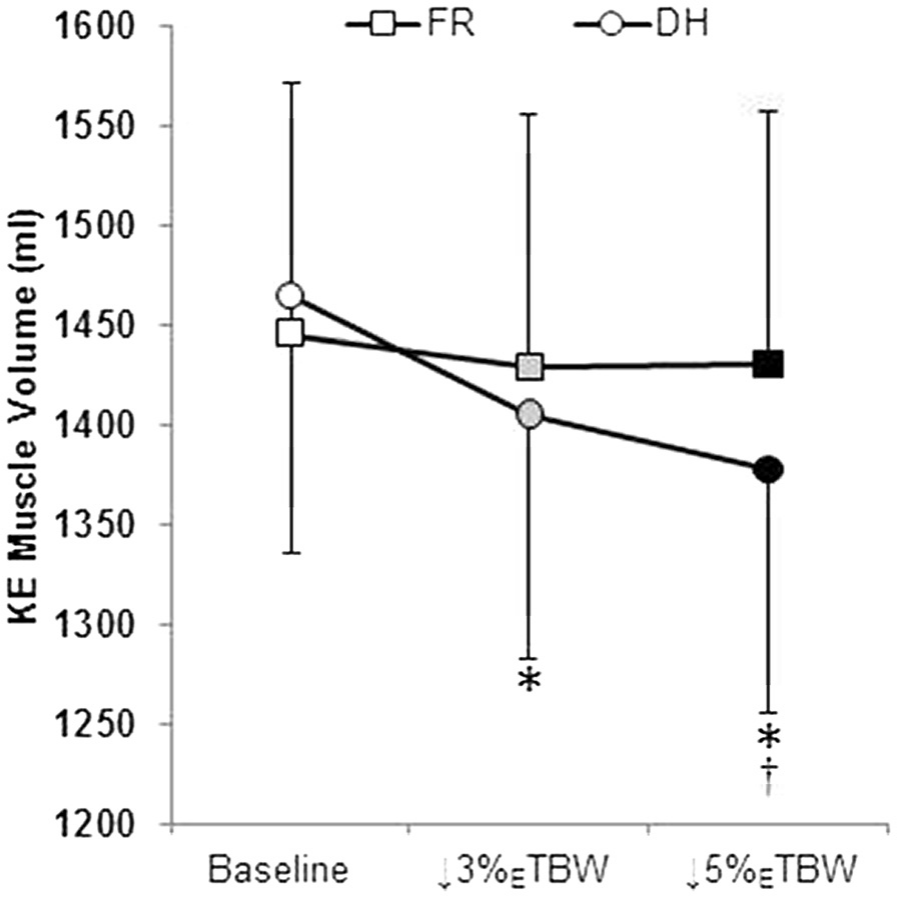
**Knee extensor muscle volume (in milliliters) in DHYD and FR.** *Significantly different from the baseline (*p* < 0.05); ^†^significantly different from FR (*p* < 0.05). FR, *squares*; DHYD, *circles*; baseline, *white fill*; ↓3% _E_TBW, *gray fill*; ↓5% _E_TBW, *black fill*.

**Figure 2:**
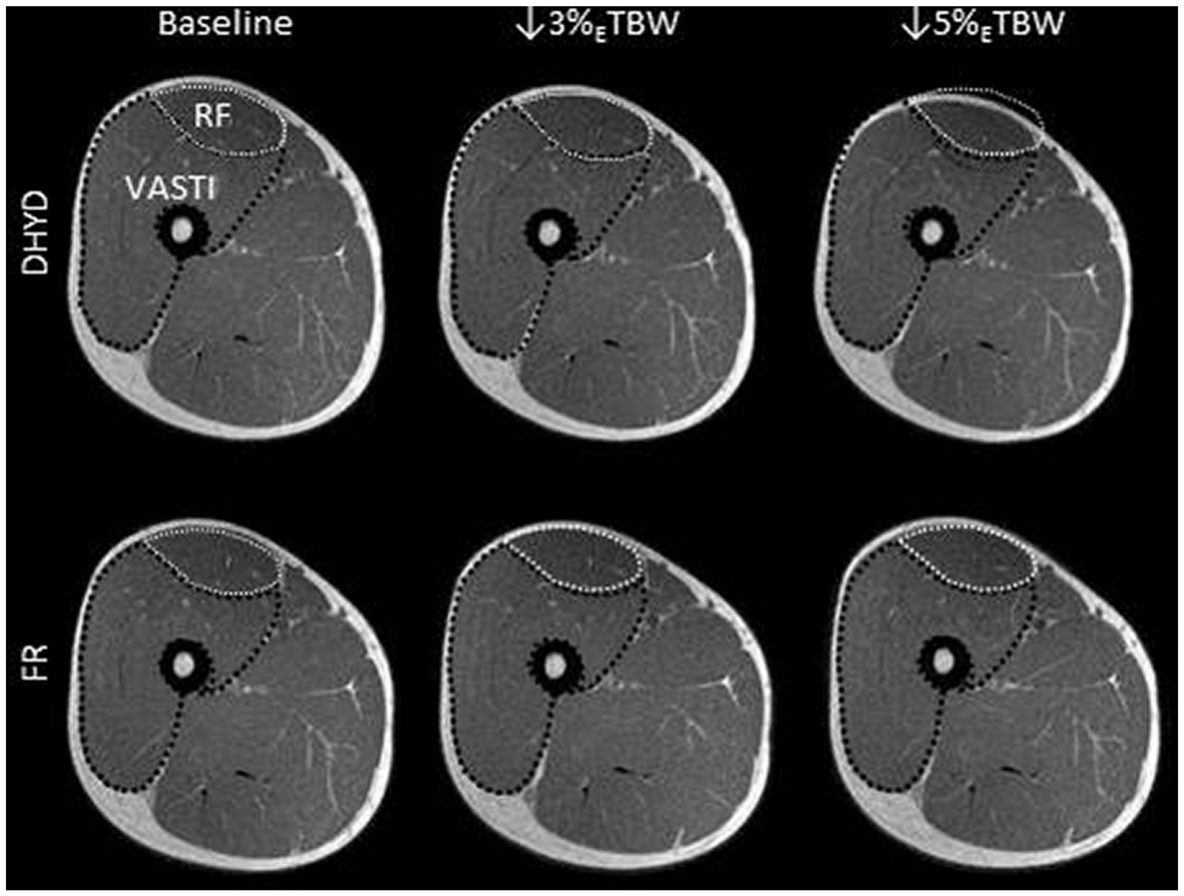
**Sample MRI slice depicting knee extensor muscle volume for DHYD and FR conditions.** RF, rectus femoris; VASTI, grouped volume of vastus lateralis, vastus intermedius, and vastus medialis. The baseline tracing of proximal muscle size is shown at ↓3% _E_TBW and ↓5% _E_TBW losses in DHYD and FR.

**Figure 3:**
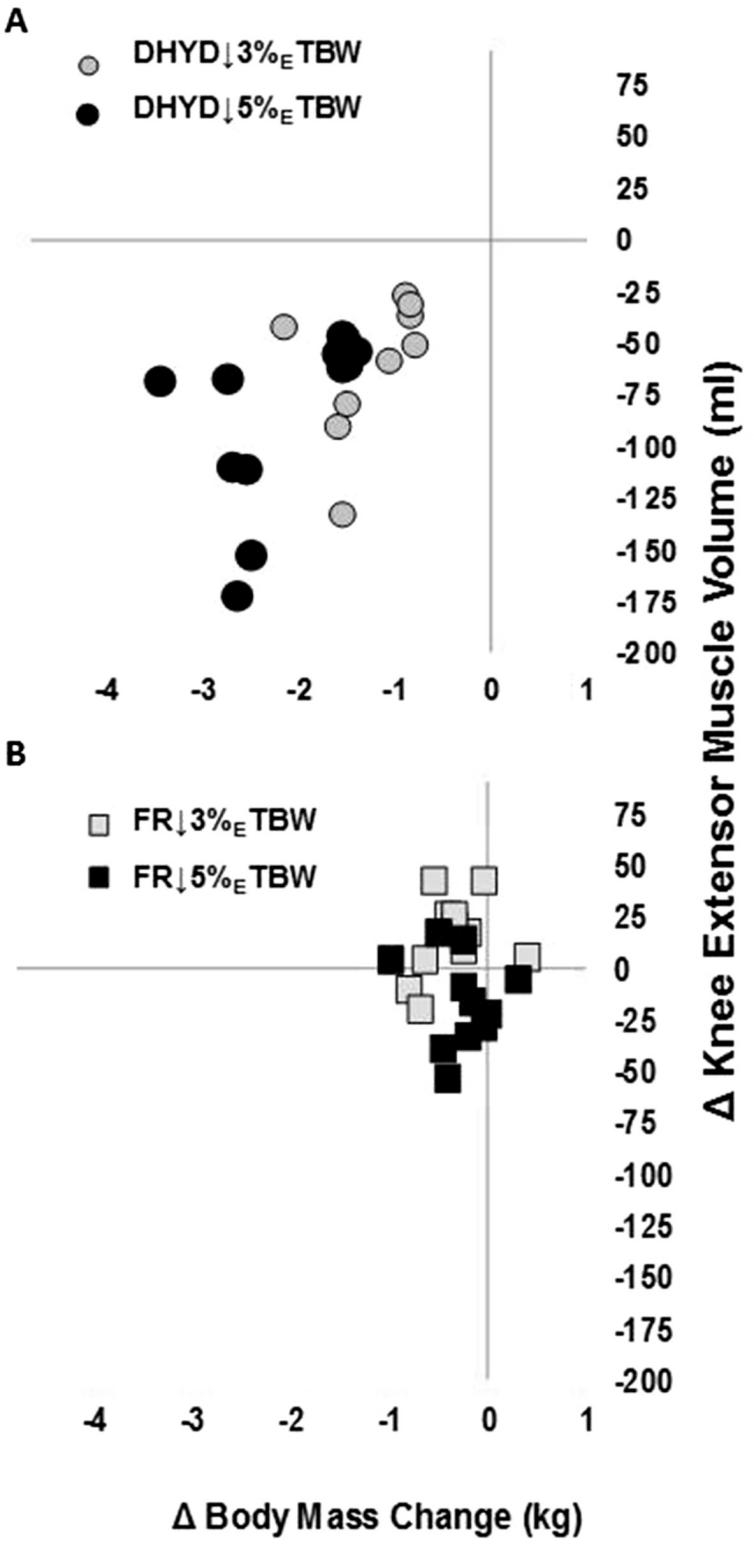
**Association between Δ body mass and Δ knee extensor volume for (A) DHYD and (B) FR.** FR, *squares*; DHYD, *circles*; ↓3% _E_TBW, *gray fill*; ↓5% _E_TBW, *black fil*.

There was a significant condition × time interaction for the knee extensor muscle volume (grouped rectus femoris and vasti muscles); *p* < 0.001, Figure 1). At baseline, there were no differences in knee extensor muscle volumes between DHYD (1,464 ± 446 ml) and FR (1,445 ± 436 ml, *p* = 0.14). In FR, there were no changes in knee extensor volume at 3% (1,430 ± 435 ml, *p* = 0.74) or 5% (1,431 ± 439 ml, *p* = 0.156) _E_TBW loss compared to that in the baseline. However, during DHYD, knee extensor volume decreased significantly at both 3% (1,406 ± 425 ml, *p* < 0.001) and 5% (1,378 ± 421 ml, *p* < 0.001) _E_TBW loss measurement points, which represented reductions of 3.96 ± 1.31% and 5.87 ± 1.97%, respectively. Knee extensor volume was lower in the DHYD compared with that in the FR at 3% (*p* = 0.005) and 5% (*p* < 0.001) _E_TBW losses. An example MRI slice from DHYD and FR conditions at each time point is shown in Figure 2. Overall, there was a significant correlation (*R* = 0.57, *R*^2^ = 0.39, *p* = 0.006) between the loss of body mass (fluid loss via sweat) and the loss of knee extensor volume during the DHYD condition. The delta change in body mass relative to the delta change in knee extensor muscle volume is displayed in DHYD (Figure 3A) and FR (Figure 3B).

When the knee extensor muscles (rectus femoris and vasti muscles) were evaluated by region across the distance of the leg in DHYD only, there were significant muscle × time point interactions in the distal (*p* < 0.001), mid-belly (*p* = 0.002), and proximal (*p* = 0.02) regions (Table 3). The volumes of the pooled vasti muscles were significantly greater than those of the rectus femoris in all regions (all *p* values < 0.001). The volume of the vasti muscles also declined significantly from the baseline to the 5% _E_TBW measurement point in the distal (*p* < 0.001), mid-belly, (*p* = 0.001), and proximal (*p* = 0.001) regions. There were no significant changes from the baseline to the 5% _E_TBW measurement point in any region of the rectus femoris (Table 3). There were no significant condition × time interactions for the biceps/triceps (*p* = 0.35) or deltoid (*p* = 0.92) muscle volumes. In addition, no main effects were observed at these sites (all *p* values > 0.05; Table 4).

**Table 3:** Regional changes in knee extensor muscle volume (milliliters) during dehydration

**Muscle(s)**	**Distal**		**Mid-belly**		**Proximal**	
	**Baseline**	**↓5%**_**E**_**TBW**	**Baseline**	**↓5%**_**E**_**TBW**	**Baseline**	**↓5%**_**E**_**TBW**
Vasti^1^	439 ± 144^†^	419 ± 139*^†^	491 ± 149^†^	460 ± 138*^†^	336 ± 110^†^	312 ± 102*^†^
RF	26 ± 9	25 ± 9	77 ± 29	73 ± 29	94 ± 33	87 ± 29*

*Significantly different vs baseline; ^†^significantly different vs RF; *p* < 0.05. ^1^Vasti, volume of the vastus lateralis, vastus intermedius, and vastus medialis; RF, rectus femoris. *n* = 11; mean ± SD.

**Table 4:** Muscle volume (milliliters) in less active musculature

**Muscle (s)**	**Condition**	**Baseline**	**↓3%**_**E**_**TBW**	**↓5%**_**E**_**TBW**
Biceps/triceps^1^	DHYD	251 ± 98	247 ± 93	249 ± 100
	FR	254 ±105	251 ± 99	259 ± 108
Deltoid	DHYD	17 ± 8	17 ± 6	17 ± 7
	FR	18 ± 7	17 ± 7	17 ± 7

^1^Combined volume of the biceps brachii and triceps. *n* = 11; mean ± SD. No significant differences were determined, *p* > 0.05.

## Discussion

The main finding of this investigation was dehydration induced through the combination of exercise and heat, which resulted in a significant reduction in skeletal muscle volume in active muscles (knee extensors). The dehydration-related loss of the knee extensor muscle volume as a result of exercise in heat was counteracted by intermitted fluid replacement. We also show for the first time that less active upper body muscles (biceps/triceps or deltoid) maintain volume when dehydration is induced by exercise in heat.

By the end of the dehydration protocol (5.75 ± 0.86% loss of _E_TBW), the decline in knee extensor muscle volume was 5.89 ±1.9%. The association (*R*^2^ = 0.32) between the change in body mass and the change in knee extensor volume suggests that muscle water was sequestered from intracellular stores of the active muscle tissue to the extracellular space. The most notable comparison to the present study is the investigation of Costill et al. [16], wherein the intracellular muscle water loss during dehydration occurred at the rate of approximately 1.20% for each 1% decrease in body mass [16]. This rate was calculated from analysis of the water content from muscle biopsies extracted from the vastus lateralis 30 min following exercise and heat dehydration protocol resulting in approximately 9% TBW loss. Applying the calculated rate established by Costill et al. [16] to the present results, yields predicted losses of knee extensor skeletal muscle water of approximately 3.31% and approximately 5.07% at each measurement point, which compares favorably to the 3.96% and 5.87% loss of knee extensor volume observed using MRI.

The knee extensors were defined as the combined volume of the vasti muscles and the rectus femoris. The vasti muscles were analyzed as a group due to the difficulty of reliably separating the vastus lateralis, vastus intermedius, and vastus medialis along the length of the leg using MRI. At baseline, the pooled vasti muscles accounted for approximately 87% of the overall knee extensor volume, and several studies suggest that these three muscles are slightly more active than the rectus femoris during cycling [25,26]. Hence, it is justifiable that larger losses (−5.89%) in volume occurred in vasti muscles compared with the rectus femoris (−5.51%). In addition, the mid-belly (−6.31%, −31 ml) and proximal regions (−7.14%, −24 ml) of the vasti muscles showed greater changes in volume compared with the distal region (−4.65%, −20 ml), which suggests differential loss of water across the length of the leg. Although this study was not designed to determine the mechanism leading to the observed loss of skeletal muscle volume, it is estimated (although debated) that 2.7 g of water are bound to each 1 g of muscle glycogen [14]. During exercise in heat, muscle glycogenolysis in the actively contracting skeletal muscle tissue is accelerated [27]; hence, water from decomplexing and that produced from metabolic pathways [28] may be free to move from intracellular into extracellular compartments.

Consistent with our hypothesis, replacement of lost fluids during exercise in heat prevented a loss of skeletal muscle volume. It was our intent to replace all fluids that were lost throughout the prolonged submaximal exercise in heat session in order to negate changes in body mass. On average, participants consumed 2,800 ± 862 ml of fluids that consisted of 164 ± 50 g of carbohydrate, 2,340 ± 718 mg sodium, 1,050 ± 323 mg potassium, and water during the FR condition. Despite our fluid replacement strategy (1.5 times fluid loss via sweat), there was a small but significant decline in body mass from the baseline at both testing time points (approximated average 0.30 kg). However, knee extensor skeletal muscle volume was preserved, suggesting no change in muscle water content when fluids are replaced during exercise in heat. Participants had a greater total exposure time in the heat chamber in FR and performed exercise at the same workload albeit at a lower heart rate. These data are in agreement with previous studies demonstrating the benefits of maintaining adequate hydration during physical exertion [29-31].

Contrary to our hypothesis, there were no changes in muscle volume in less active muscles (deltoids, biceps/triceps) during the exercise protocols. Our representation of deltoid muscle volume was small (approximately 17 ml) as it consisted five to six MRI slices superior from its appearance. Therefore, it is plausible that greater muscle volumes are required to accurately detect change via MRI. We speculate that vasoconstriction to less active muscle groups and the absence of any significant metabolic perturbations in these muscles [32] are likely to have contributed to the lack of significant change observed in the muscle volume at these sites. However, since specific physiological and metabolic measures were not conducted in this study, the contribution of intramuscular water among less active muscles during dehydration cannot be definitively established.

The time course of the changes observed during our study is worth noting. MRI scanning occurred 60 min following completion of the exercise in heat sessions to allow for equilibration of fluids across compartments [22] and to bring the metabolic rate back to near resting conditions. As a consequence to the dynamic movement of fluids between compartments during physical activity [6], it would be very difficult to accurately assess muscle water during task performance. Another limitation of the study is that water and glycogen content was not directly measured via the skeletal muscle biopsy technique. Although this would quantify water loss in both active and less actives skeletal muscles following exercise in heat, the number of muscle biopsies required from different muscles/groups would have ethical concerns. The noninvasive MRI technique used in the present study quantified muscle volume and assumed the changes of represented muscle water loss given in previous published data using the muscle biopsy technique [16].

Dehydration reduces physical work capacity, lowers tolerance to heat, and remains a significant health risk [3]. Assessment of dehydration still relies heavily on body fluid chemistry, which is impractical in many athletic or occupational settings. While compartmental fluid shifts are fundamentally important to physiological function, our findings may also have direct application to development of sensor technology aimed at detecting water content within specific tissue compartments. For instance, ultra wideband radar [33] is an emerging technology that uses relative permittivity to detect water among different tissues (muscle, fat, skin, etc) [34]. These types of sensors could play an important role in various settings including mining, space flight, and defense services.

## Conclusions

In summary, this study evaluated changes in skeletal muscle volume using MRI following exercise in heat, with or without fluid replacement. There was a significant decline in knee extensor (active muscles) volume following dehydration induced by prolonged, submaximal cycling in the heat, wherein the greatest loss of muscle volume occurred in the mid and proximal regions of the knee extensor musculature. This loss of knee extensor volume was prevented through provision of a carbohydrate-electrolyte beverage. No changes in muscle volume in the bicep/triceps brachii or deltoid (less active muscles) were detected with dehydration. The results from this study indicate that skeletal muscle volume assessed by MRI is a useful application to dehydration-related research and warrants further investigation.

## Competing interests

The authors declare that they have no competing interests.

## Authors’ contributions

KJH participated in data acquisition, completed MRI and statistical analysis, and drafted the manuscript. SBC assisted with the coordination of the study, participated in data acquisition, completed MRI analysis, and provided edits to drafts of the manuscript. TJF assisted with the coordination of the study, participated in data acquisition, and provided edits to the manuscript. LPS designed the study, secured funding, assisted with the coordination of the study, participated in data acquisition, and provided edits to drafts of the manuscript. All authors read and approved the final manuscript.
